# Aortic roadmapping during EVAR: a combined FEM–EM tracking feasibility study

**DOI:** 10.1007/s11548-024-03187-y

**Published:** 2024-06-02

**Authors:** Monica Emendi, Geir A. Tangen, Pierluigi Di Giovanni, Håvard Ulsaker, Reidar Brekken, Frode Manstad-Hulaas, Victorien Prot, Aline Bel-Brunon, Karen H. Støverud

**Affiliations:** 1 Department of Industrial Engineering, University of Tor Vergata, Rome, Italy; 2 Department of Health Research, SINTEF Digital, Trondheim, Norway; 3 HSL, Trento, Italy; 4 Department of Circulation and Medical Imaging, Norwegian University of Science and Technology, Trondheim, Norway; 5 Department of Radiology and Nuclear Medicine, St. Olavs Hospital, Trondheim, Norway; 6 Department of Structural Engineering, Norwegian University of Science and Technology, Trondheim, Norway; 7 INSA Lyon, CNRS, LaMCoS, UMR5259, Univ Lyon, 69621 Villeurbanne, France

**Keywords:** Electromagnetic tracking, Vessel-tool interaction, Aortic updated roadmap, Finite element method, Additive manufacturing, EVAR

## Abstract

****Purpose**:**

Currently, the intra-operative visualization of vessels during endovascular aneurysm repair (EVAR) relies on contrast-based imaging modalities. Moreover, traditional image fusion techniques lack a continuous and automatic update of the vessel configuration, which changes due to the insertion of stiff guidewires. The purpose of this work is to develop and evaluate a novel approach to improve image fusion, that takes into account the deformations, combining electromagnetic (EM) tracking technology and finite element modeling (FEM).

****Methods**:**

To assess whether EM tracking can improve the prediction of the numerical simulations, a patient-specific model of abdominal aorta was segmented and manufactured. A database of simulations with different insertion angles was created. Then, an ad hoc sensorized tool with three embedded EM sensors was designed, enabling tracking of the sensors’ positions during the insertion phase. Finally, the corresponding cone beam computed tomography (CBCT) images were acquired and processed to obtain the ground truth aortic deformations of the manufactured model.

****Results**:**

Among the simulations in the database, the one minimizing the in silico versus in vitro discrepancy in terms of sensors’ positions gave the most accurate aortic displacement results.

****Conclusions**:**

The proposed approach suggests that the EM tracking technology could be used not only to follow the tool, but also to minimize the error in the predicted aortic roadmap, thus paving the way for a safer EVAR navigation.

**Supplementary Information:**

The online version contains supplementary material available at 10.1007/s11548-024-03187-y.

## Introduction

Endovascular aneurysm repair (EVAR) is a minimally invasive treatment for abdominal aortic aneurysm. This procedure is traditionally guided by fluoroscopy and digital subtraction angiography for visualization of the tools and blood vessels, respectively. However, X-rays are potentially harmful both for clinicians and patients, while contrast agents are nephrotoxic [1]. The radiation and contrast doses increase along with the complexity of the procedure [2, 3]. In addition, traditional intra-operative acquisition systems are limited to bi-dimensional views. Mentally mapping the image information to 3D may be challenging and requires experience, leading to steep learning curves.

Recently, cone beam CT (CBCT) scanners and image fusion approaches have been introduced in hybrid operating rooms, allowing a reduction in contrast volume, fluoroscopy time, and procedure time in complex EVAR [4, 5]. Still, there is a need to further improve the navigation techniques by taking into account the deformations of the intra-operative anatomy, relatively to the pre-operative one, caused by the introduction of stiff wires and devices, providing clinicians a continuously updated 3D aortic map while avoiding time consuming manual registration adjustments by the operator [5, 6]. In this direction, the use of finite element methods (FEM) has been proposed to predict the above-mentioned deformations [7–9]. However, there are uncertainties related to the input parameters, e.g., angle of insertion, which can affect the results and should be further studied.

On the other side, innovative technologies for tools tracking have been explored: mainly electromagnetic (EM) tracking [10, 11] and shape sensing [12, 13]. These are promising tools that can be adopted in a clinical setting to follow the sensorized devices along their path inside the blood vessels, limiting and potentially avoiding the use of fluoroscopy [14–16]. However, by themselves these technologies do not give any direct information about the displacement of the blood vessels, hence leading to the need of contrast injections during EVAR to visualize the vessels.

**Fig. 1:**
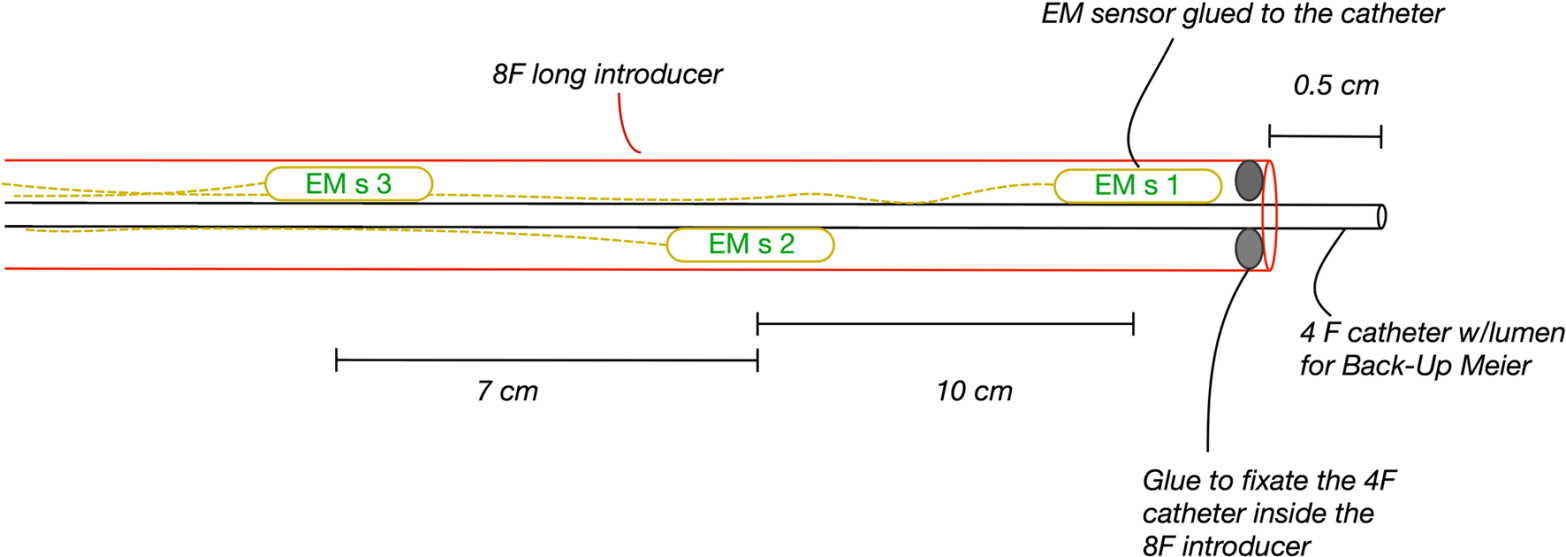
Drawing (section view) of the sensorized tool equipped with three EM sensors (EM s 1, 2 and 3, in green) glued to a 4 F (French) catheter (black), enclosed in a 8 F introducer (red). The distances between the three sensors are reported

Thus, the present work proposes to combine the two above-mentioned methods, i.e., FEM and tracking technologies, to assess whether the EM sensor data can lower the uncertainties related to the FEM predictions in a patient-specific aneurysm model. The ultimate goal is to obtain a real-time dynamic aortic roadmap during the procedure, by adjusting the predicted deformations based on the updated EM sensors’ positions, in different steps of insertion, hence helping to mitigate the radiation exposure. To the best of our knowledge, the integration of the two approaches, i.e., a finite element analysis driven by EM tracking data, has not been considered before for EVAR applications.

## Materials and methods

### Experimental setup

#### Additive manufactured model and mechanical characterization

A contrast CT scan (pixel spacing: 0.5 mm$\times$ 0.5 mm; slice thickness: 1 mm) was acquired at St. Olavs Hospital, using a Siemens Somatom CT scanner. The study was approved by the regional ethics committee (REK 2016/533) and a written informed consent was obtained. The patient was chosen for the complexity of the vascular anatomy, expected to experience large deformations due to endovascular tools insertion. The images were segmented, through a Python script by thresholding and morphological operations [17], to obtain the lumen of the aneurysmatic abdominal aorta. The root of the renal, visceral and internal iliac arteries were also segmented since their intra-operative displacement is of clinical significance. From the segmented 3D volume, cut, smoothing and re-meshing operations were carried out to obtain a suitable mesh for manufacturing. The physical model was obtained following an ad hoc lost-core casting technique. The main steps of this technique are detailed in the Online Resource. Additionally, by means of selective laser sintering technology (SLS) an ad hoc designed box with proper connectors was printed to position the model, respecting the in vivo conditions, e.g., to avoid stretching of the iliacs, during the placement.

The material used to manufacture the model was mechanically tested through an uniaxial tensile test following the ASTM D412 standard. The resulting stress–strain curve is reported in Emendi et al. [17].

**Fig. 2:**
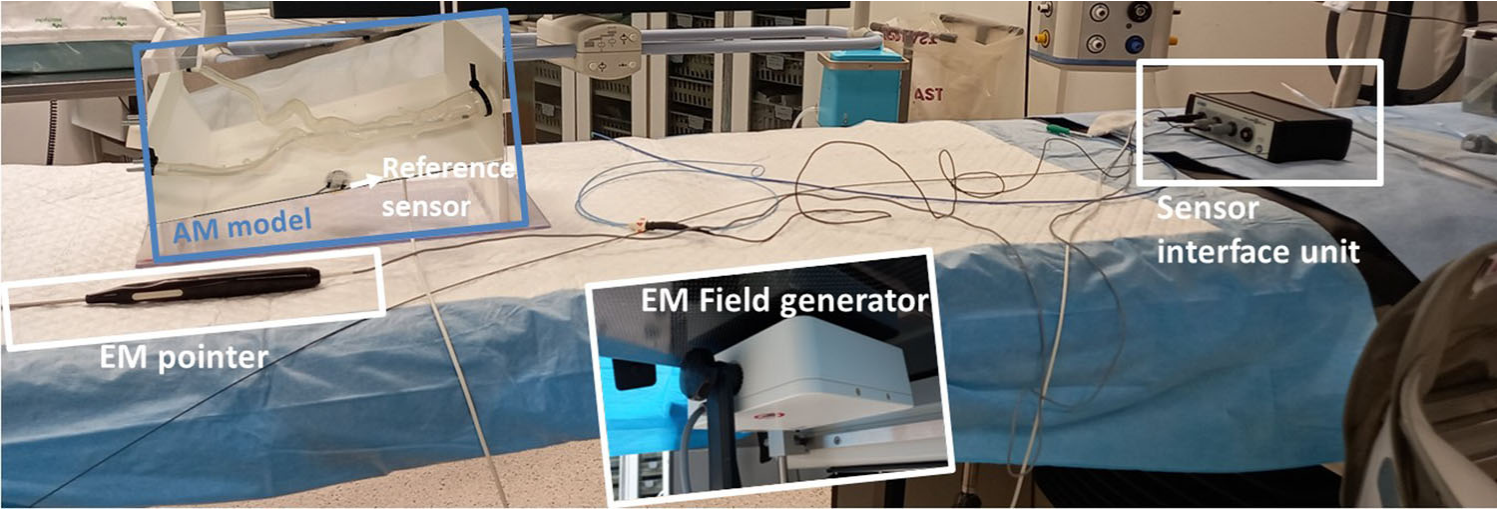
Experimental setup in the hybrid operating room with details of the EM tracking system’s components

#### Tool sensorization and mechanical characterization

A sensorized tool was manufactured as following. Three NDI Aurora (Northern Digital, Waterloo, Canada) EM sensors (5 degrees of freedom, 0.5 mm diameter $\times$ 8 mm length) were embedded in an assembled catheter at predefined distances: 0.5, 10.5 and 17.5 cm from the tip, respectively.

In detail, as shown in Fig. 1, the sensors were glued on the external surface of a 4 F catheter, which was fixated within a 8 F introducer to gather the cables of the sensors and prevent damages. This assembled tool, also referred to as sensorized catheter for simplicity, fits in a 11 F introducer that was used to stabilize the iliac access, as done in clinical practice. During the experiments, a stiff guidewire (Backup-Meier, Boston Scientific) was placed within the inner catheter.

A four-point bending test was conducted on the stiff guidewire and on the sensorized tool to retrieve the stiffness parameter needed in the numerical model. The experimental setup along with the resulting force-displacement curves obtained for the guidewire and for the sensorized tool can be found in the Online Resource. The equivalent bending stiffness of the assembled tool was obtained from the experimental data, as described in the Online Resource.

#### Experimental protocol

The experiments were conducted in a hybrid operating room equipped with a rotating C-arm scanner (Artis Zeego, Siemens, Erlangen, Germany), used to acquire CBCT images of the model at different steps of insertion. The positions of the sensors were acquired through an EM tracking system (Aurora, Northern Digital Inc, Ontario, Canada) that consists of the following parts, depicted in Fig. 2:

- An EM field generator, placed under the OR table;
- A system control unit;
- A sensor interface unit;
- A reference sensor, fixed to the box that contains the model.

The open-source software CustusX [18], designed for image-guided interventions, was used for acquisition of data from the Aurora tools, for image visualization, patient to image registration and as a graphical user interface.

**Fig. 3:**
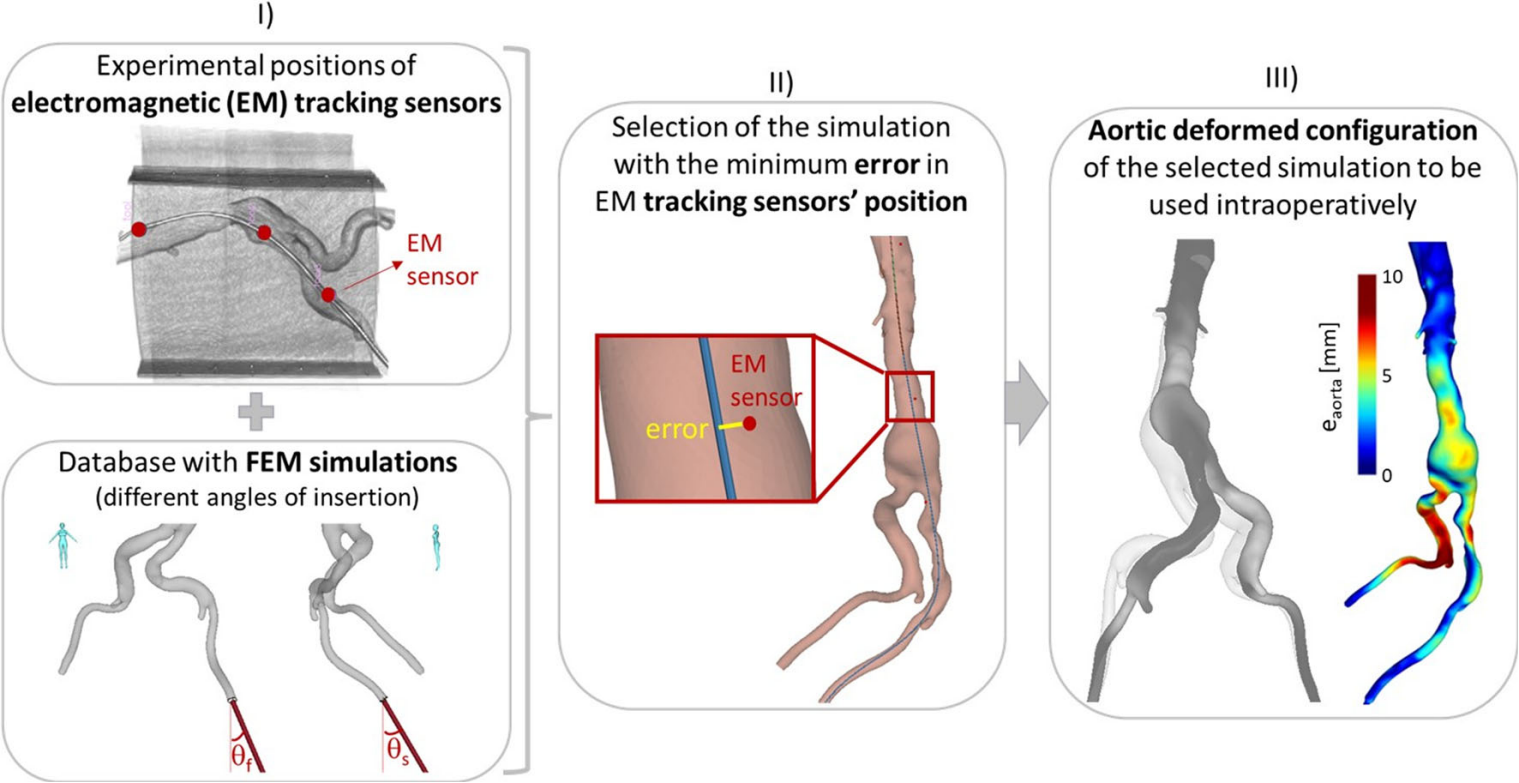
Workflow of the FEM–EM integrated approach. From left to right: (I) acquisition of EM tracking positions and creation of the database of simulation; (II) selection of the best EM fitting simulation; (III) evaluation of the aortic deformations of the chosen simulation against experimental ones, example of error map with $e_{aorta }$ values

In detail, the following experimental procedure was followed:

1. The baseline configuration of the model, placed in its box, was acquired with a rotating C-arm CBCT system and exported in DICOM format.
2. Seven tantalum radiopaque markers (0.8 mm, Tilly Medical Products AB, Lund, Sweden), fixed to the box, were sampled with the Aurora 6 DOF probe/pointer for registration of the images in the physical space (image to patient registration).
3. The image and the physical space were registered via landmark-based rigid registration (through CustusX, using the radiopaque markers).
4. A soft guidewire was inserted in the model, followed by the sensorized catheter. The soft guidewire was then removed and the stiff guidewire was pushed inside the sensorized catheter, until its floppy tip part was outside the catheter.
5. At predefined intermediate depth and at complete insertion of the stiff guidewire + sensorized catheter inside the model, the positions of the EM sensors were sampled and saved (using CustusX) and the corresponding CBCT images acquired.

### Numerical simulations

The simulations were carried out in LS-DYNA (Ansys, Canonsburg, Pennsylvania, United States), where an explicit FEM solver was adopted to calculate the aortic guidewire-induced deformations. The aorta was discretized with shell elements, with a thickness of 2 mm. Beam elements were chosen for the guidewire. An introducer with a flexible proximal part was modeled to limit the movements of the guidewire outside the vessel. The mechanical properties of the aorta and the beam were retrieved from their experimental characterization, described in the previous Sects. 2.1.1 and 2.1.2.

A velocity curve was imposed to the most distal node of the guidewire to simulate the experimental pushing action. The experimental velocity, around 40 mm/s, was increased to a maximum of 100 mm/s to save computational time, while ensuring equilibrium conditions at intermediate and final positions of interest, i.e., the kinetic vs internal energy ratio was checked to be lower than 5%. The aortic nodes in correspondence to the connectors of the boxes, to which the model was attached, were constrained in all directions. The proximal and distal extremities of the introducer were also fixed. Additional details of the numerical setup and mesh sizes are described in a previous work [17].

**Table 1:** Values of angles in the frontal and sagittal planes, $\theta _{f }$ and $\theta _{s }$ for each simulation

Simulation	$\theta _{f }$	$\theta _{s }$
$\alpha$	4^∘^	13^∘^
$\beta$	4^∘^	38^∘^
$\gamma$	23^∘^	30^∘^

**Fig. 4:**
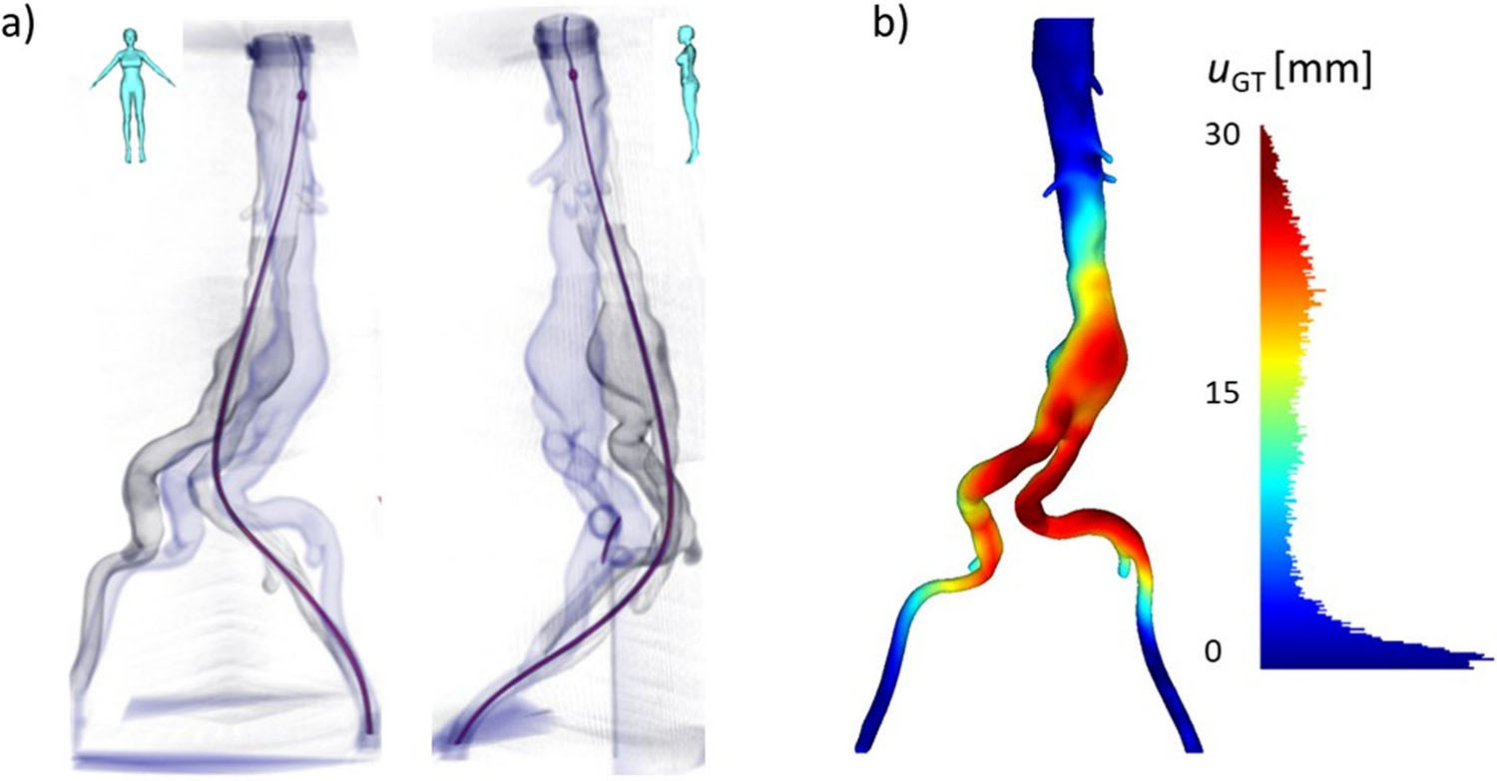
**a** Experimental deformed configuration (gray) after full insertion of the guidewire (purple), overlaid onto the baseline-undeformed configuration (blue); frontal and lateral views. **b** Color map (displayed on baseline aorta, anterior view) and corresponding histogram of the Hausdorff distance between the segmented baseline and deformed aortas, indicated as $u_{GT }$

### Setup of the FEM–EM tracking integrated approach

The workflow of the combined FEM–EM tracking approach is herein described and illustrated in Fig. 3.

1. A database of simulations with varying insertion angles, in sagittal and frontal planes, was created: three different insertion conditions were considered $\alpha$, $\beta$ and $\gamma$. Given a reference system centered in the left extremity of the model, an angle in the frontal plane, $\theta _{f }$, and one in the sagittal plane, $\theta _{s }$, were defined for each configuration, as shown in Fig. 3. The corresponding values for each simulation are reported in Table 1. The chosen values respected the given anatomical boundaries, e.g., the presence of the spine and the supine position of the patient.
2. The simulation that minimized the error in the three tracking sensors’ positions (experimental EM tracking vs numerical predicted corresponding positions), at intermediate and final steps, was automatically selected (via Python scripts) and it is later referred to as best EM fitting simulation. The above-mentioned error, $e_{sensor }$, was calculated as the average of the Euclidean distances between the experimental position of each i-th EM sensor, indicated by the vector ${\textbf {s}}_{EXP,i }$, and its numerical counterpart, indicated by the vector ${\textbf {s}}_{FEM, i }$:
  1 $$\begin{aligned} e_{\textrm{sensor}}=\frac{\sum _{i=1}^3\Vert \textbf{s}_\textrm{EXP,i}-\textbf{s}_\textrm{FEM,i}\Vert }{3}. \end{aligned}$$
3. The obtained aortic displacements of the simulations were compared to the experimental ones, to validate the approach. The experimental CBCT acquisitions were segmented using the software ImFusion (ImFusion GmbH, Munich, Germany). The experimental vs numerical differences in the deformed aortic configurations were quantified in terms of the Hausdorff distance [19] between the two sampled surfaces, indicated as $e_{aorta }$. In addition, three different regions were considered for each position, dividing the model by mid-planes between two consecutive EM sensors, as shown in Fig. 6 b). Each region was converted in its corresponding 3D volume (label map). The normalized overlap ($OV_{\textrm{P}}$) between each numerical predicted volume and its experimental counterpart was calculated as following:
  2 $$\begin{aligned} {OV_{\textrm{P}}}=\frac{V_\textrm{P}\cap V_\mathrm {EXP\_def}}{\textrm{min}({v_\textrm{P},v_\mathrm {EXP\_def}})} \times 100, \end{aligned}$$
  where $V_{\textrm{P}}$ indicates the voxels that define the numerical predicted aortic lumen of volume $v_{\textrm{P}}$, while $V_{\mathrm {EXP\_def}}$ refers to the voxels of the experimental deformed lumen of volume $v_{\mathrm {EXP\_def}}$, for each considered region. This parameter quantifies the accuracy of the FEM prediction. Moreover, the relative ground truth overlap between the experimental undeformed ($V_\mathrm {EXP\_und}$) versus deformed volumes ($V_\mathrm {EXP\_def}$) was calculated as:
  3 $$\begin{aligned} {OV_{\textrm{GT}}}=\frac{V_\mathrm {EXP\_und}\cap V_\mathrm {EXP\_def}}{\textrm{min}({v_\mathrm {EXP\_und},v_\mathrm {EXP\_def}})} \times 100, \end{aligned}$$
  this indicates the percentage of overlap between the pre- and intra- operative configuration that can be reached when considering the pre-operative 3D model for navigation purposes. The difference between two above-mentioned entities was defined as following:
  4 $$\begin{aligned} {\Delta _{\textrm{OV}}=OV_{\textrm{P}}-OV_{\textrm{GT}}}. \end{aligned}$$
  Hence, the latter parameter quantifies the improvement, in terms of overlapping volume (%) with the ground truth (experimental) deformed configuration, that can be reached using a FEM-predicted configuration instead of the undeformed one.

## Results

### Experimental results and numerical predictions

Two steps of insertion have been experimentally acquired, one intermediate, stopping just after the aortic bifurcation (Pos 1), and one final (Pos 2). The CBCTs acquired with the tools inserted at different depths were registered to the baseline CBCT (undeformed configuration) and segmented, to calculate the ground truth displacements ($u_{GT }$) in terms of Hausdorff distance. The obtained results for the final position are reported in Fig. 4.

The model was displaced mostly toward the right, in the frontal plane, and posteriorly as it can be appreciated from the sagittal view in Fig. 4. The maximum deformations were above 30 mm.

**Fig. 5:**
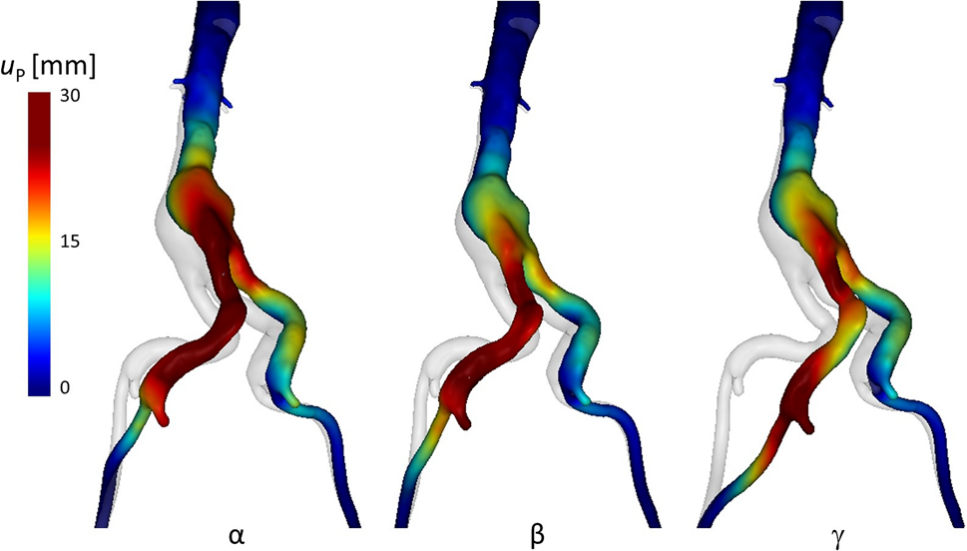
Predicted displacements, $u_{P }$, from simulations $\alpha$, $\beta$, $\gamma$, with different insertion angles. Posterior view. For each case, the color mapped final configurations (Pos 2) are overlaid onto the baseline-undeformed configuration (light gray)

Three different insertion conditions were simulated: $\alpha$, $\beta$ and $\gamma$. Focusing on the final position (Pos 2), the main differences among the three cases can be found in the way the left iliac artery (side of insertion) straightens, showing a different behavior for $\gamma$, compared to $\alpha$ and $\beta$, as displayed in Fig. 5.

### Selection of the best EM fitting simulation and validation

**Fig. 6:**
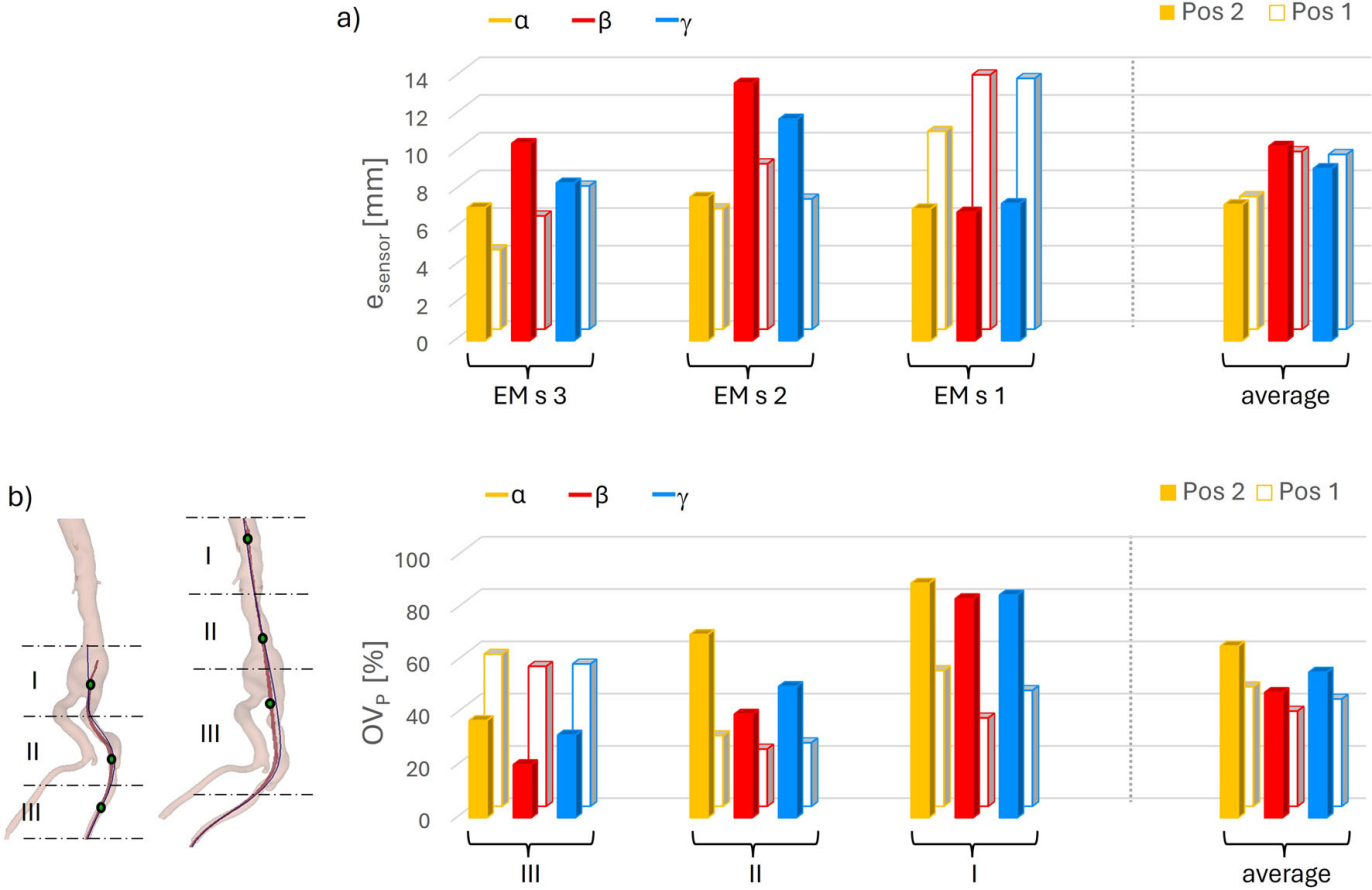
**a** Differences of EM sensors’ positions between experiments and simulations with different insertion angles ($\alpha$, $\beta$, $\gamma$) for the considered insertion steps (Pos 1, Pos 2). **b** Corresponding percentages of overlapping volumes, $OV_{\textrm{P}}$, calculated for regions I, II, III, shown on the left, for the different insertion depths, Pos 1 and 2

The numerical versus experimental errors in the positions of the EM sensors were calculated as described in Sect. 2.3 for each simulation in the database. The results are reported in Fig. 6a.

The case $\alpha$ showed the lowest errors in average and for each sensor 1, 2 and 3, hence being the best EM fitting simulation, while the greatest errors were found for $\beta$. In the considered positions, all the three sensors were always inside the model.

To assess the accuracy of the different simulations ($\alpha$, $\beta$ and $\gamma$) in the prediction of the aortic displacement, the relative overlap, $OV_{\textrm{P}}$, was measured between the numerical and experimental volumes, for the regions I, II and III, respectively linked to sensors 1, 2 and 3, as indicated in Sect. 2.3, and illustrated in Fig. 6b. The average values of $OV_{\textrm{P}}$ were 55.7%, 42.1% and 48.3% for the simulations $\alpha$, $\beta$ and $\gamma$; with the highest values obtained for $\alpha$ for all the regions (I, II and III) and all the considered positions (Pos 1 and 2). These $OV_{\textrm{P}}$ values can be commented with respect to the average value of $OV_{\textrm{GT}}$ (23.3%), which characterizes the overlap between the baseline and the deformed experimental configurations: the model indeed experienced high deformations, especially in region II for Pos 1, and regions II and III for Pos 2.

Besides, the $OV_{\textrm{P}}$ values are correlated to $e_{\textrm{sensor}}$ values, which demonstrates that, in this case, using $e_{\textrm{sensor}}$ to choose the appropriate simulation configuration yields a better aortic deformation prediction.

The values of overlap improvement (that can be reached using a FEM-predicted configuration instead of the undeformed one), $\Delta _{\textrm{OV}}$, are reported in Table 2 for each region and insertion depth.

**Table 2:** Values of $\Delta _{\textrm{OV}}$ for regions I, II and III and for positions 1 and 2

	POS 1				POS 2			
$\Delta _{\textrm{OV}}$[%]	I	II	III	Avg	I	II	III	Avg
$\alpha$	26.4	27.0	35.6	29.7	8.9	64.4	32.0	35.1
$\beta$	8.3	21.8	30.9	20.3	3.0	33.9	15.2	17.4
$\gamma$	18.8	24.2	31.8	25.0	4.5	44.5	26.5	25.2

**Fig. 7:**
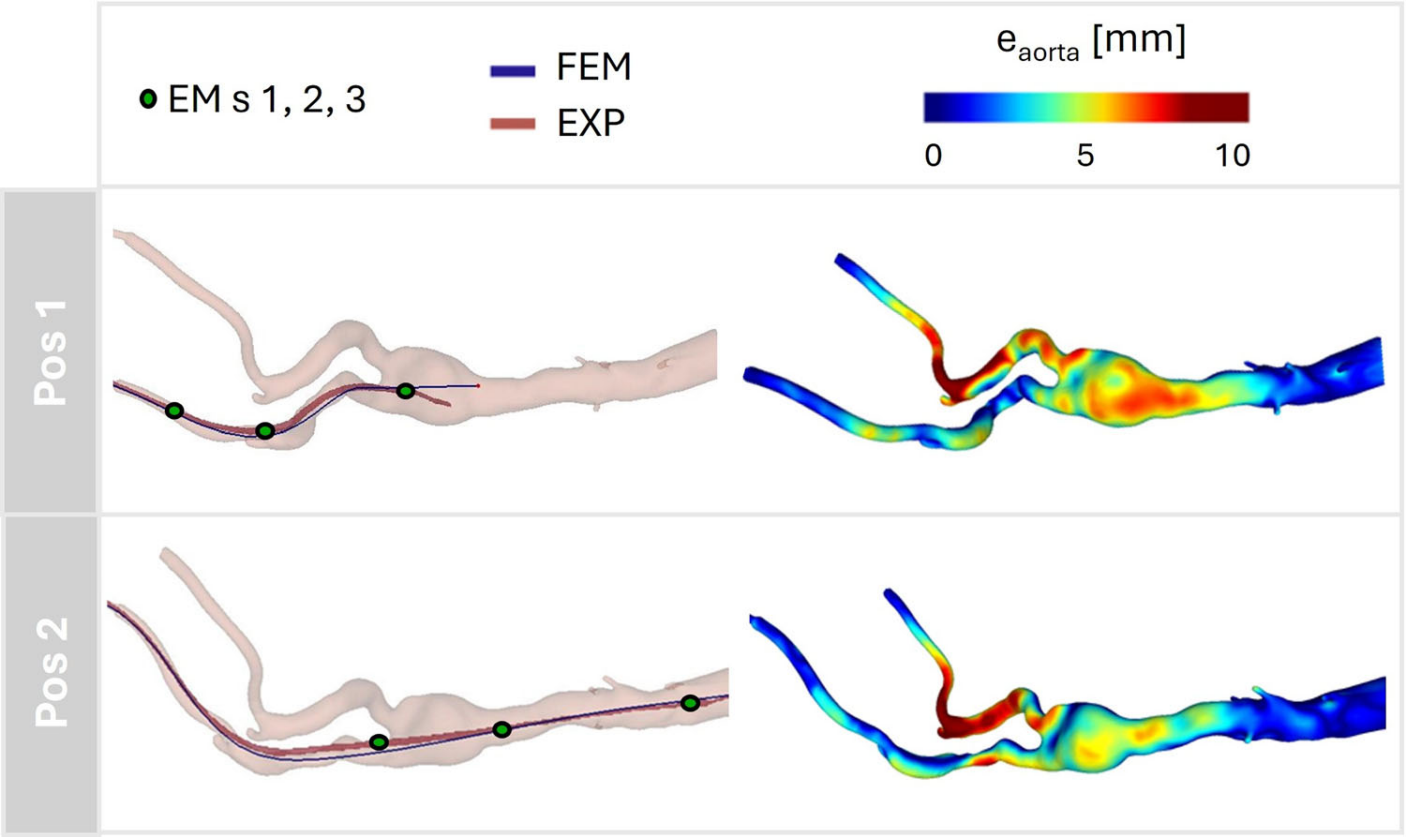
Comparison for the two considered positions (Pos 1 and 2) between the best-fitting simulation, $\alpha$ and the experiments in terms of path of the sensorized tool (left panel, in red the experimental path, from CBCT, and in blue the numerical one, from FEM) and in terms of Hausdorff distance between the deformed aortic configurations, $e_{aorta }$ (right panel). The positions of the EM sensors (EM s) 1, 2, and 3 are indicated in green (left panel)

Focusing on the final position, Pos 2, the highest value of $\Delta _{\textrm{OV}}$ was obtained in region II, where $OV_{\textrm{GT}}$ was equal to 5.65%, while $OV_{\textrm{P}}$ was 70.08%. In line with the trend of $e_{\textrm{sensor}}$, the greatest improvements were reached for $\alpha$.

The results for the best-fitting simulation, $\alpha$, in terms of comparison of tool path and aortic error, are reported in Fig. 7. For the simulation $\alpha$, the maximum error in all the positions was located at the non-cannulated iliac artery near the iliac bifurcation. At intermediate position (Pos 1), 50% of sampled nodes presented an in silico versus in vitro error below 6.7 mm, while at final position (Pos 2) 82% of the sampled nodes had an error below 9 mm.

## Discussion

We herein developed a numerical approach integrated with EM tracking data with the purpose to improve the accuracy of the FEM-predicted aortic roadmap, deformed by the guidewire insertion during EVAR. We have evaluated its feasibility and accuracy, comparing the predicted results with the experimental ground truth data, focusing on a patient-specific model.

It has to be considered that in standard clinical practice, the tools induced deformations are not taken into account; hence, the overlay error is given in this case by the difference between the pre- and intra-operative aortic configuration [5]. Our study, in line with previous ones [8, 9], shows that the FEM-predicted aortic displacements can lower this overlay error. Moreover, it demonstrates that with the integration of EM tracking data, the above-mentioned error could be further reduced.

In detail, for the studied model, the simulation that minimized the position errors of the sensors proved to be also the most accurate in the prediction of the aortic displacements. Thus, the adopted approach allowed to select the most accurate simulation (i.e., the angle of insertion that matches the experiments best), starting only from the EM sensor data, which could be acquired also in a real clinical scenario, as proposed by recent studies [20, 21]. Compared to the state of the art, this is the first work that proposes to utilize EM tracking technology not only to follow the tools in their insertion path but also to improve the FEM-derived prediction of the aortic displacements.

Another approach that aims to improve image fusion during EVAR, including correction for deformation, is the one proposed by Breininger et al. [22], which achieves this through an as-rigid-as-possible deformation energy approach using pre-operative CTs and a 2D X-ray intra-operative acquisition. This method is limited by the accuracy of the clinician when placing the landmarks and differently to our approach, it requires at least one intra-operative acquisition, and it does not involve any biomechanical consideration. Similarly, Zhang et al. [23] have proposed a novel framework able to accurately reconstruct the aortic shape by fusing a pre-operative 3D model and two intra-operative fluoroscopic acquisitions. Thus, compared to our approach, it requires a couple of acquisitions for each insertion depth of interest, making it more challenging to obtain a continuous update of the aortic roadmap.

The iliac arteries of this patient are characterized by a high tortuosity, leading to significant and complex aortic displacements. It is in challenging anatomies like the considered one that our approach is valuable, as shown by the obtained results characterized by a substantial overlap improvement.

With the adopted manufacturing procedure, it is not possible to obtain a constant thickness throughout the model, yet to limit the computational cost in the numerical counterpart a constant thickness was assigned to the model. This could lead to some mismatch between the numerical and experimental aortic displacements. Moreover, the errors between the in vitro and in silico studies could be due to the differences between the insertion steps that characterize the experimental and the numerical procedures, i.e., for simplicity and to limit the computational cost the preliminary insertion of the soft guidewire and catheter solely, were not simulated even if they were experimentally performed. Some intrinsic inaccuracies of the segmentation and registration process may contribute to the obtained in vitro vs in silico differences, along with uncertainties related to the mechanical properties of the tool and the model, the friction coefficient between them, and the maneuvers performed during the experiments of insertion that could differ from the modeled loading condition (e.g., possible torsion applied to the tool during the experiments has not been modeled).

Regarding the experimental challenges and limitations, it is worth to highlight that the sensorized tool and the model proved to be quite resistant to abrupt motions and gestures, consequent to the navigation in the highly tortuous and narrow arteries of the model.

From the obtained results, our approach seems promising to improve the intra-operative aortic roadmap also in a real clinical scenario. However, besides the necessity of clinical studies to validate it, some foreseen challenges are the integration of EM tracking technology in the surgical workflow, the cost of embedding the EM sensors in disposable instruments like catheters and guidewires along with the computational cost and the elaboration of the FEM simulations database.

## Conclusion

An innovative procedure for image fusion during EVAR, combining EM tracking technology and FEM, has been presented and experimentally evaluated. This can be considered as a proof of concept that aims to improve EVAR navigation and it may also be extended to other minimally invasive procedures, which require catheterization, e.g., treatments of structural heart diseases.

Following some suggestions on future developments are presented. Three EM sensors seemed to be enough, from the studied cases; however, a higher level of accuracy could be reached considering more positions. This might be achieved in future with the shape sensing technology [16] that can be used in combination with the EM tracking, with the capability to obtain the pose of the entire tracked tools.

On the computational side, the database of simulations, although it can be time consuming, is meant to be created before the procedure in our workflow, and then, its exploitation is compatible with intra-operative time frames; thus, the necessary time of the proposed method is compatible with non-emergency cases.

In future, to speed up the numerical part and create a larger database, a reduced order model [24] may be beneficial. For user-friendly exploitation by the clinicians, a graphical user interface might be designed on purpose, e.g., a new plugin for CustusX or 3D Slicer. The developed method could be integrated in EVAR simulators to improve their fidelity. Moreover, the experimental data gathered in our work could be used to validate alternative computational approaches to the problem.

Given that in an in vivo environment, some input variables (other than the insertion angle) carry a certain level of uncertainty, i.e., tool mechanical properties, aortic stiffness and thickness, the proposed approach could be used to select, at different insertion steps, the best-fitting simulation, that minimizes the error in terms of EM sensors’ positions, thus enhancing the accuracy of the predicted aortic roadmap.

## Supplementary Information

Below is the link to the electronic supplementary material.

Supplementary file 1 (pdf 396 KB)
