# Supplementary material for: Aortic roadmapping during EVAR: a combined FEM–EM tracking feasibility study
Source: Int J Comput Assist Radiol Surg. 2024 Jun 2;19(11):2239–47. doi: 10.1007/s11548-024-03187-y (PMC11541383; doi:10.1007/s11548-024-03187-y)
Supplement: Supplementary file 1 — (pdf 396 KB) [file 11548_2024_3187_MOESM1_ESM.pdf]

SUPPLEMENTARY MATERIAL
Aortic roadmapping during EVAR: a combined FEM-EM tracking feasibility study

Monica Emendi*[1], Geir A. Tangen[2], Pierluigi Di Giovanni[3], Håvard Ulsaker[4], Reidar Brekken[2], Frode Manstad-Hulaas[4,5], Victorien Prot[6], Aline Bel-Brunon[7], and Karen H. Støverud*[2]

[1]Department of Industrial Engineering, University of Tor Vergata, Rome, Italy
[2]Department of Health Research, SINTEF Digital, Trondheim, Norway
[3]HSL, Trento, Italy
[4]Department of Circulation and Medical Imaging, Norwegian University of Science and Technology, Trondheim, Norway
[5]Department of Radiology and Nuclear Medicine, St. Olavs Hospital, Trondheim, Norway
[6]Department of Structural Engineering, Norwegian University of Science and Technology, Trondheim, Norway
[7]Univ Lyon, INSA Lyon, CNRS, LaMCoS, UMR5259, 69621 Villeurbanne, France

*Corresponding authors.
E-mails: monica.emendi@students.uniroma2.eu, karen-helene.stoverud@sintef.no

1 Additive manufacturing procedure

The main steps of the lost-core casting technique followed to manufacture the model are herein detailed:

1. *3D printing of the patient-specific core*: the segmented lumen was offset of 0.7 mm inwards. Elongations at the extremities were added to allow the centering of the core in the mould. The obtained .stl file was printed by SLA 3500 3D printer (3D Systems, US) using Somos PerFORM (Stratasys). To improve the core's surface quality and to ease its removal, a thin layer of release agent was applied to it.

2. *Preparation of the outer mold*: the segmented lumen was offset outwards by the desired thickness of the aortic wall (2 mm). The resulting .stl was printed with the same technique used for the inner core. The value of the thickness was chosen in agreement to average data from literature[1] and previous works[2]. The printed part, called master, was used to mold the negative counterpart, i.e., the silicone outer cast (that presented injection and overflow ports for the casting phase). The latter was then finely cut in two parts, respecting the complex anatomical asymmetries.

[1]Raghavan et al. "Regional distribution of wall thickness and failure properties of human abdominal aortic aneurysm". J Biomech (2006); 39(16):3010-6.

[2]He et al. "Anthropomorphic and biomechanical mockup for abdominal aortic aneurysm". Med Eng Phys (2020) 77:60–68. Van Disseldorp et al. "Patient specific wall stress analysis and mechanical characterization of abdominal aortic aneurysms using 4d ultrasound". EJVES (2016); 52(5):635–642.

3. *Casting*: the core was centered inside the outer molds and held in position by interference fits at its extremities, the assembly was then closed through metallic pins. Two components were mixed inside a blender to obtain the molding material, i.e. PRC1719 Synthene, a transparent polyurethane resin with shore hardness A 30. The material was then poured, under vacuum, inside the assembly through the injection port. The mold was left cure inside an industrial oven (at 70° of for 2h 30min).

4. *Core removal*: the outer mold was opened and the inner core removed by mechanically breaking it into pieces.

2 Four-point bending test of the sensorized tool

To retrieve the mechanical properties of the ad-hoc sensorized tool, to be used in the numerical setup, a four-point bending test was conducted. The experimental setup and the force-displacement curves obtained for the Back-Up Meier guidewire and the catheter, used for the experimental acivities, are illustrated in Fig. S1.

To obtain the Young's modulus, $\mathrm{E_{cath,wire}}$, from the experimental data the following equation, derived from the beam theory, was applied:

$$\mathrm{E_{cath,wire}} = \frac{\mathrm{k_{cath,wire}} a^2(3s+2a)}{12\mathrm{I_{cath,wire}}}, \tag{S1}$$

where $\mathrm{k_{cath,wire}}$ are the slopes of the experimental curves, a is the distance between the support and the actuator=14mm, s is the span between the actuators=22mm, $\mathrm{I_{cath,wire}}$ are the moment of inertia, equal to:

$$\mathrm{I_{wire}} = \frac{\pi \mathrm{d_{wire}}^4}{64}, \tag{S2}$$

$$\mathrm{I_{cath}} = \frac{\pi(\mathrm{d_e}^4 - \mathrm{d_i}^4)}{64}, \tag{S3}$$

where $\mathrm{d_{wire}}$ is the diameter of the guidewire, $\mathrm{d_e}$ is the external diameter of the catheter and $\mathrm{d_i}$ is the internal diameter of the catheter.

Assuming for simplicity that the assembled tool can be modeled with a full circular cross section, the equivalent bending stiffness of the assembled tool (guidewire inserted inside the catheter), following Shadmehri et al.[3], was obtained as:

$$\mathrm{E_{eq}I_{eq}} = \mathrm{E_{wire}I_{wire}} + \mathrm{E_{cath}I_{cath}}. \tag{S4}$$

[3]Shadmehri, Derisi, and Hoa. "On bending stiffness of composite tubes." Composite structures 93.9 (2011): 2173-2179.

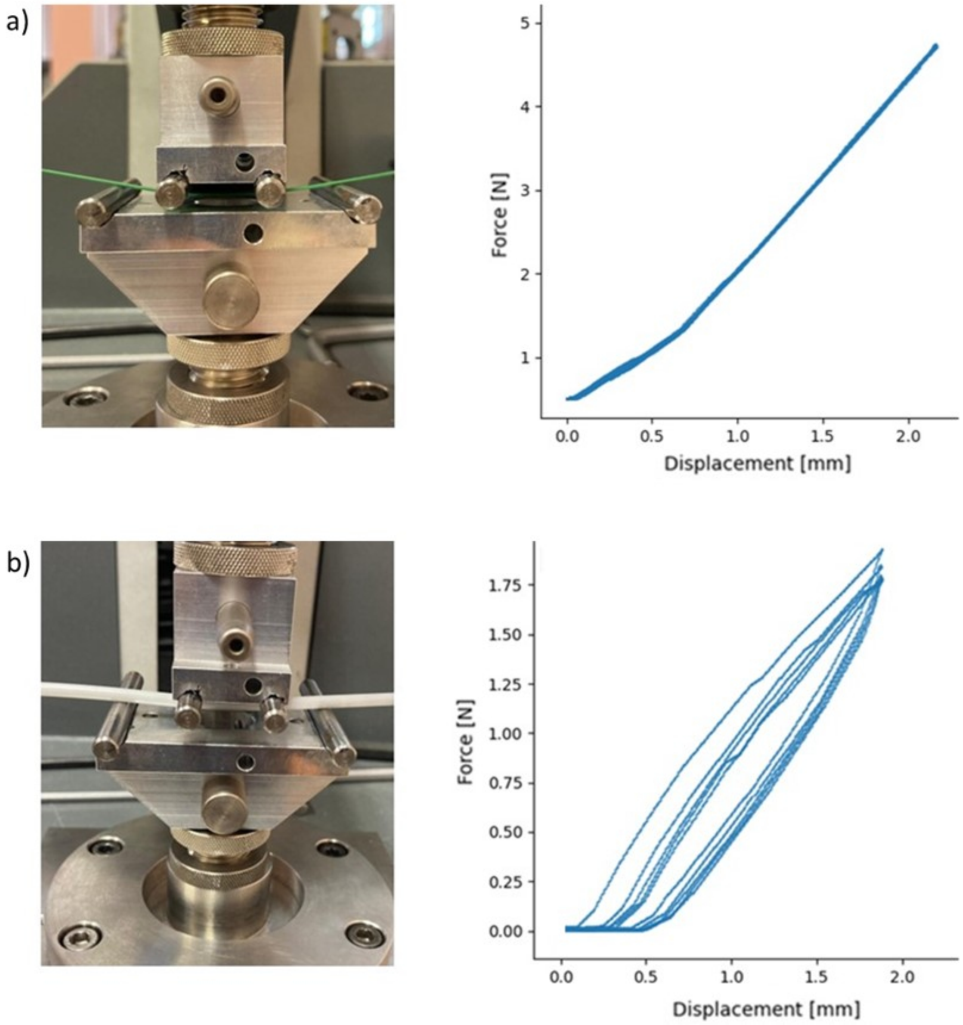

Figure S1: a) Four-point bending test of the Back-Up Meier guidewire and corresponding force-displacement curve; b) Four-point bending test of the sensorized catheter and corresponding force-displacement curve.
